# Vocal Cord Monitoring by Flexible Fiberoptic Laryngoscopy During Thyroid Radiofrequency Ablation

**DOI:** 10.1089/ve.2023.0012

**Published:** 2023-09-25

**Authors:** Roberto Valcavi, Francesca Gaino, Roberto Novizio, Giuseppe Mercante

**Affiliations:** Department of Endocrinology, Interventional Thyroidology, Endocrine and Thyroid Clinic, Reggio Emilia, Italy.; Department of Biomedical Sciences, Humanitas University, Pieve Emanuele, Milan, Italy.; Department of Otorhinolaryngology-Head and Neck Surgery, IRCCS Humanitas Research Hospital, Rozzano, Milan, Italy.; Interventional Thyroidology, Endocrine and Thyroid Clinic, Reggio Emilia, Italy.

**Keywords:** thyroid nodule, radiofrequency ablation, flexible fiberoptic laryngoscopy, recurrent laryngeal nerve, hydrodissection

## Abstract

***Introduction:*** Thermal injury to recurrent laryngeal nerve (RLN) during radiofrequency ablation (RFA) can produce temporary or permanent vocal cord paralysis.^1^ Hydrodissection with cold 5% glucose of “danger triangle” protects RLN during RFA.^2^ When RFA is performed under local anesthesia, RLN function is monitored by patients producing vocal sounds.^3^ Large lesions requiring longer RFAs warrant general sedation where voice cannot be assessed, therefore, an additional technique for RLN protection is advisable. Observation of passive symmetrical vocal cord movements during breathing by laryngeal ultrasonography is useful in assessing vocal cord function^4^; however, flexible-fiberoptic fibrolaringoscopy (FFL) is gold standard for assessing vocal cord movements,^5^ anticipating potential RLN damage. We report FFL monitoring during RFA under general sedation on a large thyroid nodule. FFL during RFA may detect RLN irritation and dysfunction if asymmetry in passive vocal cord movements is noted. Should asymmetry appear, RFA operator stops delivering energy and repositions electrode needle.

***Materials and Methods:*** Thyroid function tests, blood glucose, creatinine, transaminase, International-Normalized-Ratio, and electrocardiogram were performed. Operating room (OR) layout created sufficient space for ear-nose-throat (ENT) and RFA operators. An examination with a fiberscope camera demonstrated normal vocal cord adduction during phonation and abduction during breathing. The procedure was assisted by an anesthetist administering fentanyl 50 mcg, midazolam 1.5 to 5.0 mg, and propofol infusion 2 mg/(kg·h). General sedation was conducted so that reflexes were attenuated but still observable. Incorporating in OR by an anesthetist who performs general sedation reduces side effects and complications.^6^ Ultrasonography showed a 34-mL right lobe nodule abutting on the RLN area. After sedation with propofol, the ENT specialist inserted an endoscope until the glottic plane. During calm breathing, vocal cords moved symmetrically. After obtaining anterior nodule hydrodissection from strap and sternocleidomastoid muscles with 10 mL of 2% lidocaine, posterior hydrodissection was achieved by ultrasound-guided administration of 30 mL of 5% cold glucose. Anterior and posterior hydrodissections merged, separating nodule from neck structures. The radiofrequency electrode needle was then inserted into the nodule, initially positioned in inferior nodule portion adjacent to danger triangle previously isolated by hydrodissection. Initial power was 30 watts. Moving-shot technique was used.

***Results:*** FFL was performed throughout thyroid RFA. Symmetric vocal cord movements during breathing demonstrated no RLN irritation. FFL monitoring allowed observation of natural reflexive phenomena, including swallowing. Complete nodule ablation was achieved. FFL performed post-RFA confirmed normal vocal cord motility.

***Conclusions:*** We report the first-time use of FFL for vocal cord monitoring during RFA. FFL was easily performed by the ENT specialist and well tolerated by the patient. Avoiding danger triangle and precise RFA needle positioning is key in preventing RLN injury. Benign nodules regrow if total ablation is not achieved^7^ and some authors propose additional procedures to complete ablation^8^ that obviously incurs costs. Total RFA nodule ablation-assisted FFL monitoring eliminates the need for repetitive RFAs, thus reducing overall treatment costs. Finally, FFL monitoring does not prolong procedure, as it is performed simultaneously with RFA. FFL is a valid technique when used in conjunction with hydrodissection to further prevent RLN thermal injury during RFA, especially indicated for large thyroid nodule ablation and professional voice users.

***Patient Consent and Permission:*** The patient provided written consent for FFL monitoring and permission to use his portrayals and ultrasonographic images during RFA. The study was completed in accordance with the Declaration of Helsinki as revised in 2013. Adherence to institutional review board protocols was granted.

***Disclaimer:*** Representation of any instrumentation within the video does not indicate any endorsement of the product and/or company by the publisher, the American Thyroid Association, or the authors.

No competing financial interests exist.

Runtime of video: 9 mins 39 secs

## Graphical abstract

**Figure:**
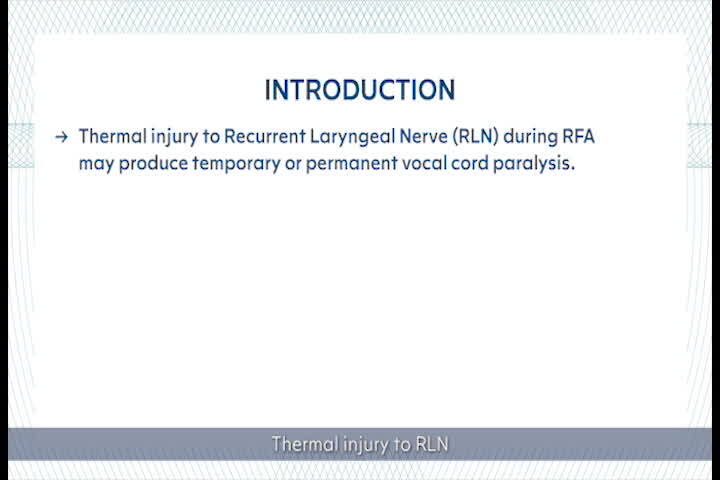

**Figure:**
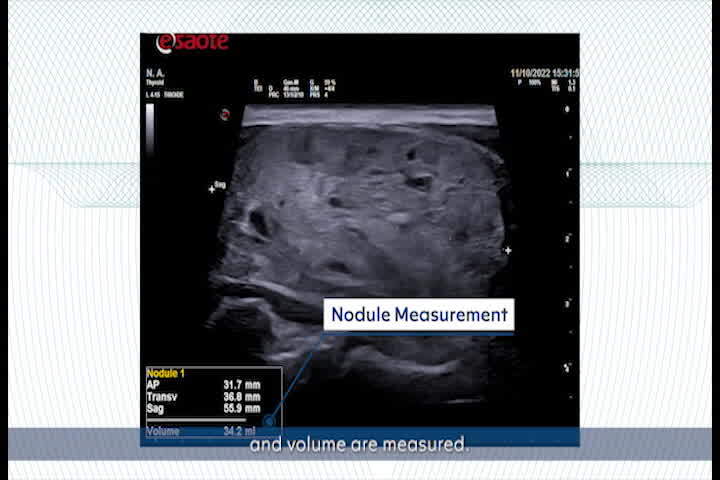

**Figure:**
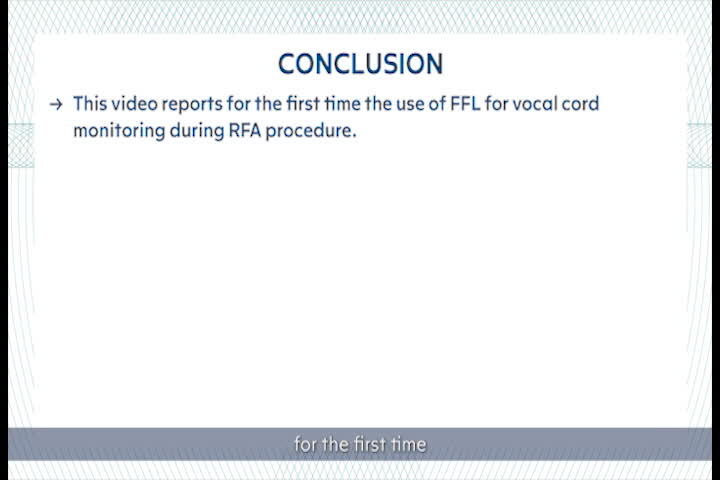

## Video



## Files

**PDF:** 
